# Re-administration of platinum-based chemotherapy for recurrent endometrial cancer: an ancillary analysis of the SGSG-012/GOTIC-004/Intergroup study

**DOI:** 10.1007/s10147-024-02585-1

**Published:** 2024-07-13

**Authors:** Shoji Nagao, Shin Nishio, Kazuhiro Takehara, Shinya Sato, Toyomi Satoh, Muneaki Shimada, Satoshi Yamaguchi, Hiroshi Tanabe, Masashi Takano, Kouji Horie, Yuji Takei, Yuichi Imai, Yumi Hibino, Kosei Hasegawa, Munetaka Takekuma, Kazuto Nakamura, Hirokuni Takano, Keiichi Fujiwara, Hisashi Masuyama

**Affiliations:** 1 Department of Obstetrics and Gynecology, Okayama University Graduate School of Medicine, Dentistry and Pharmaceutical Sciences, Okayama University, 2-5-1 Shikata-cho, Kita-ku, Okayama, 700-8558 Japan; 2 Department of Obstetrics and Gynecology, Kurume University School of Medicine, 67 Asahi-machi, Kurume, Japan; 3 Department of Gynecologic Oncology, NHO Shikoku Cancer Center, 160 Minami Umenomoto, Matsuyama, Japan; 4 Department of Obstetrics and Gynecology, Tottori University, 36-1 Nishi-cho, Yonago, Japan; 5 Department of Obstetrics and Gynecology, Institute of Medicine, University of Tsukuba, 1-1-1 Tennodai, Tsukuba, Japan; 6 Department of Gynecology, Tohoku University Hospital, 1-1 Seiryo-machi, Aoba-ku, Sendai, Japan; 7 Department of Clinical Biobank, Tohoku University Advanced Research Center for Innovations in Next-Generation Medicine, 1-1 Seiryo-machi, Aoba-ku, Sendai, Japan; 8 Department of Medical Oncology, Hyogo Cancer Center, 13-70 Kitaoji-cho, Akashi, Japan; 9 Department of Obstetrics and Gynecology, Jikei University School of Medicine, 3-9-18 Nishishinbashi, Minato-ku, Tokyo, Japan; 10 Department of Obstetrics and Gynecology, National Defense Medical College, 3-2 Namiki, Tokorozawa, Japan; 11 Department of Gynecologic Oncology, Saitama Cancer Center, 780 Oazakomuro, Ina-machi, Kitaadachi-gun, Saitama, Japan; 12 Department of Obstetrics and Gynecology, Jichi Medical University, 3311-1 Yakushiji, Shimotsuke, Japan; 13 Department of Obstetrics and Gynecology, Yokohama City University Hospital, 3-9 Fukuura, Kanazawa-ku, Yokohama, Japan; 14 Department of Gynecologic Oncology, Saitama Medical University International Medical Center, 1397-1 Yamane, Hidaka, Japan; 15 Department of Gynecology, Shizuoka Cancer Center, 1007 Shimonagakubo, Nagaizumi-cho, Sunto-gun, Shizuoka, Japan; 16 Department of Gynecology, Gunma Prefectural Cancer Center, 617-1 Takabayashinishi-machi, Ota, Japan

**Keywords:** Recurrent endometrial cancer, Re-administration of platinum-based chemotherapy, Platinum-free interval, Secondary platinum response

## Abstract

**Background:**

We previously demonstrated the applicability of the concept of “platinum sensitivity” in recurrent endometrial cancer. Although immune checkpoint inhibitors have been widely incorporated into endometrial cancer treatment, the debate continues regarding treatment options in patients with recurrent endometrial cancer who have previously received platinum-based chemotherapy. In this study, we assessed the duration of response to secondary platinum-based treatment using pooled data from the SGSG-012/GOTIC-004/Intergroup study.

**Methods:**

Among the 279 participants in the SGSG-012/GOTIC-004/Intergroup study wherein platinum-based chemotherapy was re-administered for managing recurrent endometrial cancer between January 2005 and December 2009, 130 (47%) responded to chemotherapy. We compared the relationship between platinum-free interval and duration of secondary platinum-based treatment using pooled data.

**Results:**

In 40 patients (31%), the duration of response to secondary platinum-based treatment exceeded the platinum-free interval. The duration of response to secondary platinum-based treatment exceeded 12 months in 51 patients (39%) [platinum-free interval: < 12 months, 14/48 (29%); 12–23 months, 18/43 (42%); 24–35 months, 8/19 (42%); ≥ 36 months, 11/20 (55%)]. In particular, in eight patients (6%), the duration of response to secondary platinum-based treatment exceeded 36 months [platinum-free interval: < 12 months, 3/48 (6%); 12–23 months, 0/19 (0%); 24–35 months, 2/19 (11%); ≥ 36 months, 3/20 (15%)].

**Conclusions:**

Re-administration of platinum-based chemotherapy for recurrent endometrial cancer may result in a long-term response exceeding the platinum-free interval in some patients. Even in the current situation, where immune checkpoint inhibitors have been introduced, re-administration of platinum-based chemotherapy is worth considering.

## Introduction

The concept of “platinum sensitivity” in patients with epithelial ovarian cancer was introduced by Markman et al. in 1991 and has since been widely accepted [1]. This concept is based on evidence that the effectiveness of the re-administration of platinum-based chemotherapy is dependent on the platinum-free interval (PFI), which is defined as the period between the completion of platinum-based chemotherapy and subsequent disease recurrence. Patients with recurrent ovarian cancer and PFI of > 6 months are classified as “platinum sensitive.” In previous studies, these patients usually received platinum-based chemotherapy. The response rate ranged between 27 and 65% and the median survival period was 12–24 months [1, 2]. On the other hand, patients with a PFI of < 6 months are classified as “platinum resistant”. In these patients, the chemotherapy response rate was 10–30% with a median survival period of 6–9 months. “Platinum sensitivity” has been effectively considered a basic concept for estimating the prognosis and selecting treatments of recurrent ovarian cancer. However, in recent years, maintenance therapy using bevacizumab and/or poly-(adenosine diphosphate-ribose) polymerase (PARP) inhibitors has been introduced as a treatment strategy for advanced epithelial ovarian cancer. The concept of “platinum sensitivity” is no longer considered very effective in the management of recurrent cancer [3].

The retrospective exploratory SGSG-012/GOTIC-004 Intergroup study showed that the concept of “platinum sensitivity” can be applied to recurrent endometrial cancer as well as epithelial ovarian cancer [4]. In that study, as the PFI increased from < 6, 6–11, 12–23, and ≥ 24, the response rate increased to 25%, 38%, 61%, and 65%, respectively. Furthermore, progression-free survival (PFS) and overall survival (OS) increased with an increase in the PFI. Between < 12 and ≥ 12 months of PFI, there were significant differences in the median PFS (4.4 months vs. 10.3 months, *p* < 0.0001) and median OS (13.8 months vs. 40.9 months, *p* < 0.0001).

Recently, the effectiveness of the immune checkpoint inhibitor pembrolizumab and the multikinase inhibitor lenvatinib in the management of recurrent endometrial cancer has been confirmed. These drugs are widely used in clinical practice [5, 6]. In addition, regarding treatment of recurrent endometrial cancer, deciding whether to re-administer platinum-based regimens or use lenvatinib plus pembrolizumab combination chemotherapy (LEN/PEM) is necessary. The median PFS in women with a PFI of ≥ 12 months who were re-administered platinum-based therapy in the SGSG-012/GOTIC-004 Intergroup study was 10.3 months, which was likely equivalent to or even better than that afforded by LEN/PEM [among those with cancers deficient in the MMR protein (dMMR): 10.7 months, among those having cancers with intact MMR protein expression (MMR proficient, pMMR): 6.6 months] [4, 6]. Currently, reconsidering the positioning of platinum re-administration for recurrent endometrial cancer is necessary. However, other than the SGSG-012/GOTIC-004 Intergroup study, there are a few large-scale reports on platinum re-administration for recurrent endometrial cancer [4].

We considered re-analyzing the pooled data of the SGSG-012/GOTIC-004 Intergroup study [4]. In particular, this study aimed to focus on the duration of secondary platinum response (DSPR), which had not been analyzed in previous study. Since the data set of the study did not include data on the best response date, we used the DSPR definition by Markman et al. instead of the duration of response [7].

## Patients and methods

Consecutive patients with histologically confirmed endometrial cancer or uterine carcinosarcoma who received second-line platinum-based chemotherapy between January 2005 and December 2009 were enrolled in the SGSG-012/GOTIC-004/Intergroup study. Chemotherapy administered concurrently with radiotherapy (RT) is not regarded as platinum-based chemotherapy, even if it includes a platinum agent.

Among the 279 participants in the SGSG-012/GOTIC-004/Intergroup study, 130 (47%) responded to second-line platinum-based chemotherapy. The relationship between the PFI and DSPR was evaluated using pooled data. PFI was defined as the period from the last date of platinum-based therapy administration using first-line chemotherapy to the date of the diagnosis of recurrence. DSPR was defined as the period from the start date of second-line platinum-based chemotherapy to the date of subsequent radiological relapse or progression or to the date of the last contact for disease-free patients. The probability of DSPR was estimated using the Kaplan–Meier method. The estimated DSPRs were compared using log-rank test. GraphPad Prism version 10.2.3 was used to construct survival curves and comparison.

The SGSG-012/GOTIC-004/Intergroup study was conducted according to the principles of the Declaration of Helsinki and approved by the institutional review board of each participating institution. This ancillary analysis was conducted with the approval of the Ethics Committee of the Okayama University Hospital (approval no. 2311-004). No new data were collected in this study.

## Results

### PFI and response to second-line chemotherapy

Overall, 279 patients from 30 centers were registered in the SGSG-012/GOTIC-004/Intergroup study. Table 1 presents the relationship between the PFI and response rate. The response rates among patients wherein the PFI was < 12, 12–23, 24–35, and ≥ 36 months were 31% (48/153), 64% (43/67), 68% (19/28), and 65% (20/31), respectively. Overall, 130 women (47%) who responded to second-line platinum-based chemotherapy participated in the ancillary analysis.

**Table 1:** Platinum-free interval and response to second-line chemotherapy (*N* = 279)

Response	Platinum-free interval (months)			
	PFI < 12	12 ≤ PFI < 24	24 ≤ PFI < 36	PFI ≥ 36
Complete response	18	19	11	10
Partial response	30	24	8	10
Stable disease	33	8	2	5
Progressive disease	67	10	4	5
Not evaluable	5	6	3	1
Total	153	67	28	31
Overall response (%)	31	64	68	65

PFI: platinum-free interval

### Patient characteristics

Table 2 summarizes the major patient characteristics and their tumors. The International Federation of Gynecology and Obstetrics stage at diagnosis was III/IV in 96 patients (74%). Endometrioid carcinoma accounted for 70% in all patients. More than two-third were grade 1 or 2. Approximately two-third of the patients received taxanes plus carboplatin as first-line chemotherapy. Furthermore, approximately 80% of the patients received taxanes plus carboplatin as second-line chemotherapy.

**Table 2:** Patient characteristics (*N* = 130)

	*N* (%)
FIGO stage at the time of the primary therapy	
I	20 (15)
II	14 (11)
III	60 (46)
IV	36 (28)
Histology	
Endometrioid	91 (70)
Grade 1	22
Grade 2	40
Grade 3	25
Not determined	4
Serous	12 (9)
Adenosquamous	5 (4)
Clear cell	3 (2)
Carcinosarcoma	15 (12)
Others	4 (3)
Regimen of chemotherapy	
First-line chemotherapy	
Cisplatin based	
AP	29 (22)
Others	5 (4)
Carboplatin based	
TC	82 (63)
DC	2 (2)
Others	10 (8)
Nedaplatin based	2 (2)
Second-line chemotherapy	
Cisplatin based	
AP	12 (9)
Others	6 (5)
Carboplatin based	
TC	86 (66)
DC	20 (15)
Others	4 (3)
Nedaplatin based	2 (2)

AP: doxorubicin plus cisplatin, TC: paclitaxel plus carboplatin,

DC: docetaxel plus carboplatin

### Relationship between PFI and DSPR

**Fig. 1:**
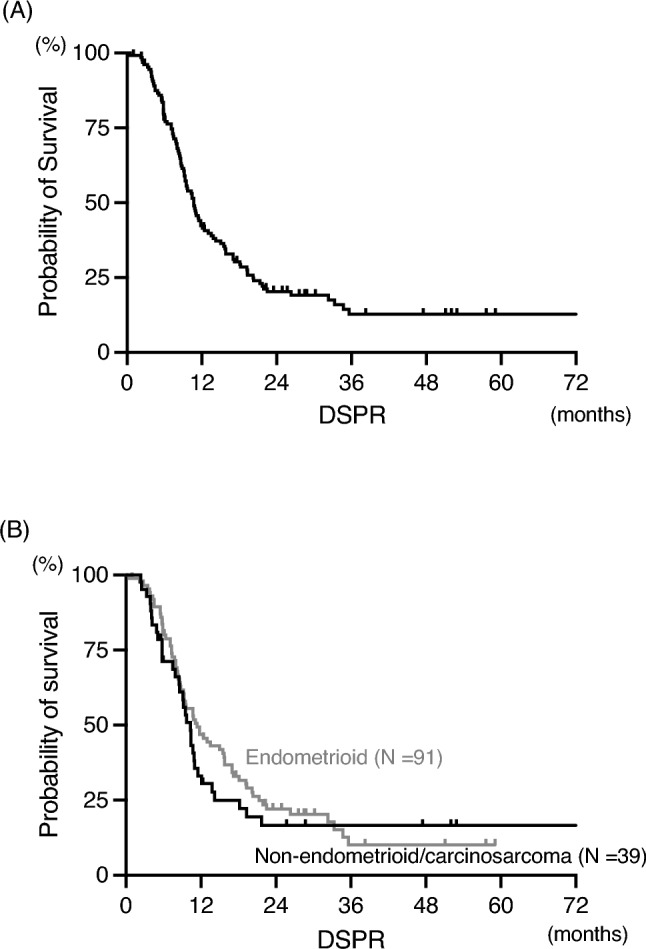
**A** Duration of secondary platinum response in all participants. The median duration of secondary platinum response is 11.2 months. **B** Duration of secondary platinum response by histological type. Endometrioid histology (gray line) includes grade 1 to 3 endometrioid carcinoma (*N* = 91). Non-endometrioid/carcinosarcoma histology (black line) includes serous, adenosquamous, clear cell, carcinosarcoma, and other histological types (*N* = 39). Median duration of secondary platinum response is 11.4 and 10.3 months, and hazard ratio is 0.855 (95% confidence interval is 0.557 to 1.312, *p* = 0.457). DSPR: Duration of secondary platinum response

Figure 1A shows the estimated DSPR in all participants who responded to second-line platinum-based chemotherapy. The median DSPR was 11.2 months, and the survival rates at 12, 24, and 36 months were 46.8, 20.1, and 12.3%, respectively. There was no significant difference in DSPR between endometrioid histology (*n* = 88) and non-endometrioid histology including carcinosarcoma (*n* = 42) (Fig. 1B).

**Fig. 2:**
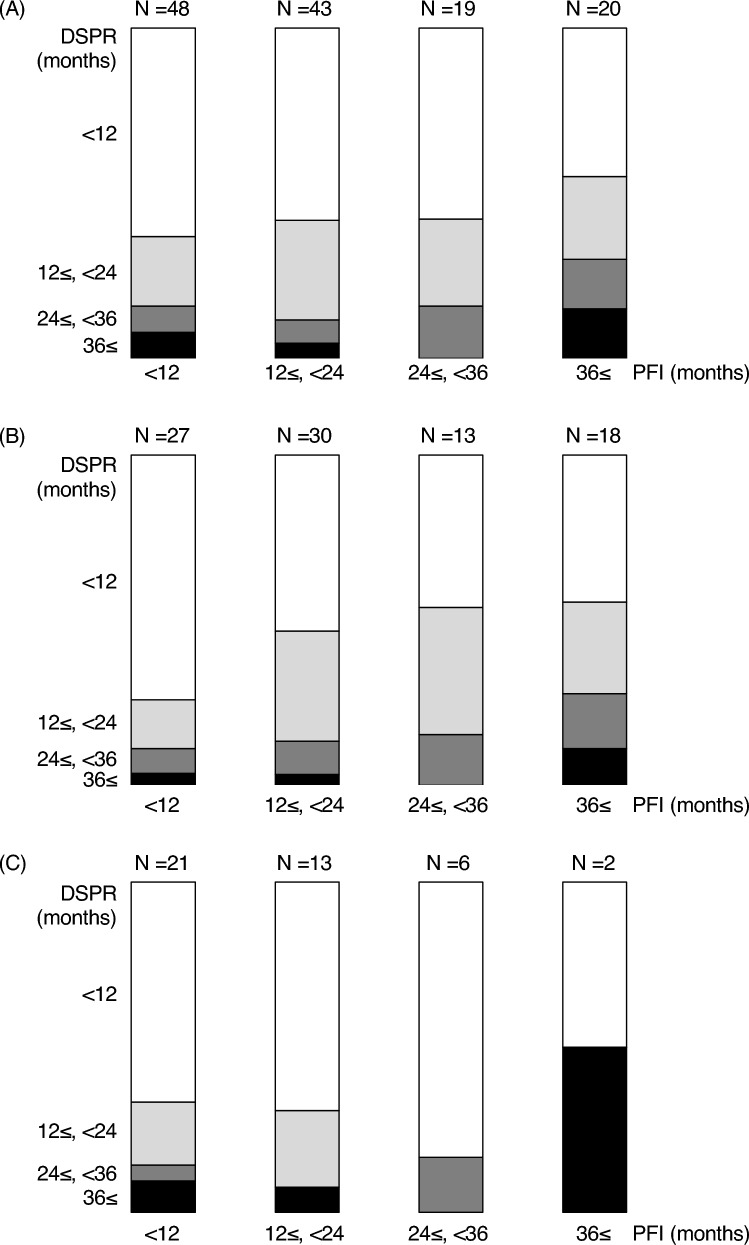
**A** The relationship between platinum-free interval and duration of secondary platinum response in whole patients who respond to second-line platinum-based chemotherapy. **B** The relationship between platinum-free interval and duration of secondary platinum response in endometrioid histology. **C** The relationship between platinum-free interval and duration of secondary platinum response in non-endometrioid/carcinosarcoma. PFI: platinum-free interval, DSPR: duration of secondary platinum response

Figure 2A shows the relationship between PFI and DSPR in 130 patients who responded to second-line platinum-based chemotherapy. Overall, the DSPR tended to increase as the PFI increased. However, this trend was not clear, and there were quite a few patients wherein the DSPR exceeded the PFI. This tendency was similar even when limited to endometrioid histology (Fig. 2B). On the other hand, considering non-endometrioid histology including carcinosarcoma, there were few cases of long PFI; the trend was unclear (Fig. 2C).

Table 3 lists the details of PFI and DSPR. The numbers of patients wherein the PFI was < 12, 12–23, 24–35, and ≥ 36 months and the DSPR exceeded the PFI were 28/48 (58%), 10/43 (23%), 1/19 (5%), and 1/20 (5%), respectively. The DSPR was > 12 months in 51 patients [39%; PFI: < 12 months, 14/48 (29%); 12–23 months, 18/43 (42%); 24–35 months, 8/19 (42%); and ≥ 36 months, 11/20 ([55%)]. In particular, 8 patients (6%) had a DSPR of > 36 months [PFI: < 12 months, 3/48 (6%); 12–23 months, 0/19 (0%); 24–35 months, 2/19 (11%); and ≥ 36 months, 3/20 (15%)].

**Table 3:** Relationship between the duration of secondary platinum response and platinum-free interval in patients who responded to second-line platinum-based chemotherapy

PFI (months)	DSPR	*N*	PFI < DSPR
	(months)		*N*
< 12	< 12	34	14
	12 ≤, < 24	8	8
	24 ≤, < 36	3	3
	36 ≤	3	3
12 ≤, < 24	< 12	25	
	12 ≤, < 24	13	5
	24 ≤, < 36	3	3
	36 ≤	2	2
24 ≤, < 36	< 12	11	
	12 ≤, < 24	5	
	24 ≤, < 36	3	1
	36 ≤	0	0
36 ≤	< 12	9	
	12 ≤, < 24	5	
	24 ≤, < 36	3	
	36 ≤	3	1

(*N* = 130)

PFI: platinum-free interval, DSPR: duration of secondary platinum response

### Clinical factors influencing the DSPR (Table 4)

**Table 4:** Duration of secondary platinum response in subgroups (*N* = 130)

Subgroups	*N*	Median DSPR (months)	Hazard ratio 95% CI	*p*
Histology				
Endometrioid	91	11.4	0.855	0.457
Non-endometrioid/carcinosarcoma	39	10.3	0.557–1.312	
Age				
< 65 years	70	10.9	0.944	0.773
≥ 65 years	60	10.3	0.638–1.397	
Primary FIGO stage				
I/II	34	9.3	1.152	0.536
III/IV	96	10.9	0.720–1.841	
Previous RT				
Yes	9	5.8	1.793	0.087
No	121	10.7	0.743–4.324	
PFI				
≥ 12 months	80	11.8	0.737	0.131
< 12 months	50	9.3	0.485–1.121	
Site of recurrence				
Distance/Abdomen	90	10.7	1.174	0.457
Pelvis/LN	40	10.9	0.776–1.776	
Chemotherapy for recurrence				
TC/DC	107	10.7	0.971	0.907
Non-TC/DC	23	9.7	0.592–1.592	
Changes of chemotherapy				
Yes	57	9.7	1.071	0.729
No	73	10.8	0.724–1.585	

DSPR: duration of secondary platinum response, RT: radiation therapy, PFI: platinum-free interval, LN: lymphnode, TC: paclitaxel plus carboplatin, DC: docetaxel plus carboplatin

No significant clinical factors that might have affected the DSPR were identified. The DSPR may have been shortened in patients with a history of RT. However, the number of patients who had received RT was quite few and details were unclear.

Table 5 presents the first- and second-line chemotherapies in patients with the DSPR exceeding the PFI. Among 40 patients, 12 patients received paclitaxel plus carboplatin combination therapy for both first-line and second-line chemotherapies; the remaining 28 patients received different chemotherapy.

**Table 5:** First- and second-line chemotherapy for patients with the duration of secondary platinum response exceed platinum-free interval (*N* = 40)

First-line regimen	Second-line regimen	*N*
AP	TC	6
	DC	3
DP	TC	1
TC	AP	5
	TC	12
	DC	10
	AC	2
	CPT-P	1

DSPR: duration of secondary platinum response PFI: platinum-free interval, AP: doxorubicin + cisplatin, DP: docetaxel + cisplatin, TC: paclitaxel + carboplatin, DC: docetaxel + carboplatin

AC: doxorubicin + carboplatin, CPT-P: irinotecan + cisplatin

## Discussion

In the SGSG-012/GOTIC-004/Intergroup study, 47% of the patients with recurrent endometrial cancer responded to the re-administration of platinum-based therapy. Among these responders, the DSPR exceeded the PFI in 31% of the patients. In particular, the DSPR exceeded 12 months in 39% of the cases. Furthermore, the DSPR lasted for ≥ 36 months in 6% of the cases. Thus, re-administration of platinum-based chemotherapy for recurrent endometrial cancer is expected to produce long-term effects in a significant number of patients.

Several studies have revealed that secondary responses to platinum-based chemotherapy in patients with recurrent ovarian cancer are highly dependent on PFI [1, 2, 8–11]. Second-line platinum-based regimens are expected to be effective in a longer period, the longer the PFI. A retrospective review by Markman et al. revealed that the DSPR rarely exceeded the PFI [7]. Only four (3%) out of 121 patients with assessable secondary responses had DSPR of longer duration than the prior response period in their study [7]. The SGSG-012/GOTIC-004/Intergroup study revealed that the PFI is a predictor of response and survival after second-line platinum-based chemotherapy in patients with recurrent endometrial as well as in ovarian cancers [4]. However, the DSPR exceeded the PFI in 40 (31%) of 130 patients with recurrent endometrial cancer. PFI may not determine DSPR as clearly in patients with recurrent endometrial cancer as in those with ovarian cancer. Endometrial cancer has a lower rate of homologous recombination deficiency compared with epithelial ovarian cancer. The mechanisms by which platinum drugs responded to may differ [12, 13]. As presented in Table 5, more than two-third of the patients whose DSPR exceeded the PFI received different first- and second-line chemotherapy regimens. However, differences in chemotherapy regimens administered at initial treatment and recurrence had no clear effect on the DSPR (Table 4).

We could not identify significant clinical factors that might have influenced the DSPR (Table 4). Although this study included many patients with a high-risk type of histology, such as carcinosarcoma and non-endometrioid, we did not find any effect of the histologic type on the DSPR. In addition, the regimen of chemotherapy administered did not have a clear effect on the DSPR. On the other hand, although there were only 9 patients with previous RT, they had very short DSPR, suggesting that RT might have had negative effects on the DSPR.

In the LEN/PEM group in the KEYNOTE-775 trial, responses were observed in 33.8% of patients. The median PFS period for whole patients was 7.3 months [6, 14]. Based on the shape of the PFS curve, it was estimated that slightly less than 20% of the patients would have very long-term recurrence-free survival. A certain number of patients are expected to have a very long survival period or cure with LEN/PEM. On the other hand, the shape of the survival curve of the DSPR (Fig. 1A) in our study reveals a flat tail. Re-administration of platinum-based chemotherapy can also result in very long survival or cure in slightly less than 20% of patients with recurrent endometrial cancers. In addition, as many as 39% of the patients had a response longer than 12 months, 6% of the patients had a response longer than 36 months. Similar to that with LEN/PEM, re-administration of platinum-based chemotherapy may afford very long-term survival in a certain number of patients.

The final analysis of the ENGOT-En9/LEAP-001 phase III randomized controlled study, which directly compared the efficacy and safety between LEN/PEM and paclitaxel and carboplatin combination therapy (TC) as a first-line chemotherapy for endometrial cancer, revealed that LEN/PEM did not significantly extend PFS or OS [15]. However, LEN/PEM prolonged PFS and OS in the subgroup of dMMR (the hazard ratios were 0.61 and 0.57, respectively). In addition, LEN/PEM significantly prolonged PFS and OS in patients with previous neoadjuvant/adjuvant chemotherapy (the hazard ratios were 0.52 and 0.64, respectively), which comprised approximately 15% of whole study participants. Unfortunately, the results by PFI in this study have not been disclosed. On the other hand, according to the real-world data from the US, there was no significant difference between the response to platinum-based chemotherapy and LEN/PEM re-administration in patients with recurrent endometrial cancer who had previously received platinum-based chemotherapy [16]. Patients with PFI ≤ 12 months had a 44.1% and 46.5% response to LEN/PEM and platinum-based chemotherapy, respectively; patients with PFI > 12 months had a 75.0% and 66.7% response, respectively. As observed in the ENGOT-En9/LEAP-001sudy, in patients with dMMR, LEN/PEM may offer longer survival than TC therapy, and there are no clear criteria for the re-administration of platinum-based chemotherapy for the treatment of recurrent endometrial cancer [15]. In future, it is expected that the criteria for treatment selection will be clarified by carefully collecting clinical information. The Japanese Gynecologic Oncology Group is currently collecting real-world data to clarify treatment strategies for recurrent endometrial cancer (JGOG2055s study: UMIN000050897).

This study has a few limitations. The patients in the SGSG-012/GOTIC-004/Intergroup study received treatment of recurrence between 2005 and 2009 [4]. Although the standard primary treatment of endometrial cancer has not changed, the social backgrounds of patients may differ. It cannot be denied that this could have had some influence on the treatment outcomes. In addition, the data collected in this study did not include the date of response confirmation; therefore, calculating the duration of the response was impossible. Therefore, the comparability with data from recent clinical trials, including those on immune checkpoint inhibitors, has decreased. Furthermore, data regarding the expression status of mismatch repair genes and microsatellite instability were not included. Although there are some problems, as described above, there is no other large-scale, properly cleaned dataset of patients with recurrent endometrial cancer who received platinum-based chemotherapy, which does not undermine the significance of this analysis.

Re-administration of platinum-based chemotherapy for recurrent endometrial cancer might result in a long-term response beyond the PFI. Even in the current situation where immune checkpoint inhibitors have been introduced for management, re-administration of platinum-based chemotherapy is worth considering.

## Data Availability

The data that support the findings of this study are not openly available due to reasons of sensitivity and are available from the corresponding author upon reasonable request.
